# Embracing the *impact* from instrumented mouthguards (iMGs): A survey of iMG managers' perceptions of staff and player interest into the technology, data and barriers to use

**DOI:** 10.1002/ejsc.12101

**Published:** 2024-03-19

**Authors:** Gregory Roe, Sarah Whitehead, Lindsay Starling, David Allan, Matt Cross, Éanna Falvey, Simon Kemp, Cameron Owen, Clint Readhead, Danielle Salmon, Sean Scantlebury, Keith Stokes, Greg Tierney, James Tooby, Ross Tucker, Ben Jones

**Affiliations:** ^1^ Carnegie Applied Rugby Research (CARR) Centre Carnegie School of Sport Leeds Beckett University Leeds UK; ^2^ World Rugby Dublin Ireland; ^3^ Centre for Health and Injury and Illness Prevention in Sport University of Bath Bath UK; ^4^ UK Collaborating Centre on Injury and Illness Prevention in Sport (UKCCIIS) University of Bath Bath UK; ^5^ Sport and Exercise Sciences Research Institute Ulster University Belfast UK; ^6^ Premiership Rugby London UK; ^7^ School of Medicine & Health University College Cork Cork Ireland; ^8^ Rugby Football Union Twickenham UK; ^9^ London School of Hygiene and Tropical Medicine London UK; ^10^ England Performance Unit Rugby Football League Manchester UK; ^11^ South Africa Rugby Union Cape Town South Africa; ^12^ Division of Physiological Sciences and Health through Physical Activity, Lifestyle and Sport Research Centre Department of Human Biology Faculty of Health Sciences University of Cape Town Cape Town South Africa; ^13^ School of Behavioural and Health Sciences Faculty of Health Sciences Australian Catholic University Brisbane Queensland Australia

**Keywords:** athlete health, concussion, head acceleration event, technology adoption

## Abstract

Instrumented mouthguards (iMGs) are a novel technology being used within rugby to quantify head acceleration events. Understanding practitioners' perceptions of the barriers and facilitators to their use is important to support implementation and adoption. This study assessed men's and women's rugby union and league iMG managers' perceptions of staff and player interest in the technology, data and barriers to use. Forty‐six iMG managers (men's rugby union and league *n* = 20 and *n* = 9 and women's rugby union and league *n* = 7 and *n* = 10) completed an 18‐question survey. Perceived interest in data varied across staff roles with medical staff being reported as having the most interest. The iMG devices were perceived as easy to use but uncomfortable. Several uses of data were identified, including *medical applications, player monitoring* and *player welfare.* The *comfort, size and fit of the iMG* were reported as the major barriers to player use. *Time constraints* and a *lack of understanding of data* were barriers to engagement with the data. Continued education on how iMG data can be used is required to increase player and staff buy‐in, alongside improving comfort of the devices. Studies undertaken with iMGs investigating player performance and welfare outcomes will make data more useful and increase engagement.

## Highlights

- From the perspective of instrumented mouthguard (iMG) managers (appointed practitioners responsible for the collection and analysis of iMG data in sports teams), the iMG technology is easy to use and has important sport science, medical and player welfare applications, yet is rarely used for these purposes in applied practice.
- To overcome barriers to adoption, technology companies should work closely with athletes to optimise the comfort, size and fit of iMGs and look to address any technological shortcomings perceived by practitioners, such as robustness and data anomalies.
- Future research should focus on the use of iMGs for sport science, medical and welfare purposes to improve understanding of iMG data and guide practice.

## 1 INTRODUCTION

Collision sport athletes are at an increased risk of head injuries (Gardner et al., 2014, 2015; West et al., 2021) with concussion incidence ranging from 15.5 to 20.9 per 1000 match‐hours in men's rugby league and union (Eastwood et al., 2023; West et al., 2021) and 2.8–10.3 concussions per 1000 match‐hours in women's rugby league and union (King et al., 2022; Starling et al., 2023). Governing bodies are proactively trying to reduce both concussion and head acceleration events (HAEs) (Eliason et al., 2023; Hendricks et al., 2023). HAEs occur from both direct (i.e. direct head impacts) and indirect (i.e. inertial loading from contact with the body) impacts (Tierney, 2021). Quantifying the frequency, magnitude and mechanisms of HAEs can inform player welfare initiatives. Furthermore, evaluating interventions aimed at reducing HAEs can determine the success of player welfare initiatives (Jones et al., 2022; Tierney, 2021) both at a policy and practice level (Hendricks et al., 2023).

Various technologies are available that have been designed to approximate in vivo HAEs outside of laboratory settings. These consist of inertial sensors embedded in wearables, such as headbands, helmets, skull caps, skin patches and mouthguards (Le Flao et al., 2022). However, technologies not fixed to the skull suffer from excessive displacement, inaccurate HAE counts and acceleration magnitudes (Press & Rowson, 2017). Thus, due to their coupling to the skull, instrumented mouthguards (iMGs) have shown the most promise for accurately approximating HAE in the field (Wu et al., 2016).

Prior to the implementation of any new technology in sport, the validity and reliability of the instruments must be considered alongside their usefulness and ability to integrate into practice (Torres‐Ronda & Schelling, 2017; Windt et al., 2020). The construct and criterion validity of four different iMG systems have been recently established (Jones et al., 2022). For example, laboratory validation of iMGs designed and manufactured by Prevent Biometrics (Minneapolis, MN, USA Laboratory) yielded a concordance correlation coefficient of 0.984 (95% CI: 0.977–0.989), while field‐based video verification analysis yielded a positive predictive value of 0.94 (0.92–0.95) and a sensitivity value 0.75 (0.67–0.83) during on‐field video verification validation (Jones et al., 2022). Additionally, the fit (85% [range 67%–100%] perceived no issues with fit), comfort (perceived comfort had a median 8 out of 10 [interquartile range 7–8]) and function (67% [range 44–94]) of the iMGs was reported by players, whilst practitioners reported on the usability (using the system usability scale) of data preparation (83.8 out of 100 [range 53–95]) and management (80.0 out of 100 [range 50–98]) (Jones et al., 2022). However, the study was limited by the small sample of rugby league practitioners and players evaluating the iMGs over a relatively short period of time. Now that iMGs are much more widely used within practice, further consideration of the feasibility can be evaluated. Specifically, further understanding of the contextual factors that impact their implementation (e.g. rugby union and rugby league men's and women's cohorts) is important to optimise adoption (Bauer & Kirchner, 2020; Windt et al., 2020). Considering the differences in environments by code and sex (e.g. funding and professionalism) (Scantlebury et al., 2022), context‐specific perceptions of the devices should be considered.

The introduction of a new technology in any environment requires a change in attitudes (i.e. thoughts about and feelings towards the new technology) and behaviours (i.e. how the technology is integrated into existing ways of doing (Wong et al., 2023)). Successful adoption is often suggested to be the result of a balance between the perceived usefulness and ease of use of the technology along with social and environmental factors. These combine to influence behavioural intention and use behaviour (Holden & Karsh, 2010; Momani, 2020). Thus, for policymakers in rugby, it is important to understand the factors that influence iMG technology adoption before making policy decisions pertaining to its implementation.

Given the ability of iMGs to provide data on HAE and therefore inform player welfare initiatives, there have been recent attempts by governing bodies (e.g. World Rugby, Rugby Football League and Rugby Football Union) to systematically promote and implement widespread adoption of iMGs at the elite level. Within each club or environment where the iMG devices have been implemented by the governing bodies, there has been an appointed practitioner (i.e. the ‘iMG manager’). The iMG manager was responsible for the collection and analysis of their respective team's iMG data. However, anecdotally, there has been variable uptake and use of iMGs across and between competitions. To support the future use of iMGs, environment‐specific (i.e. code and gender) practitioners' perceptions should be investigated to provide insight into the barriers and facilitators of implementation (Bauer & Kirchner, 2020). Therefore, this study firstly aims to investigate the iMG managers' perspective on staff and player interest and use of the technology and data. Secondly, it aims to identify the iMG managers' perceived barriers to adoption of iMG devices in practice.

## 2 MATERIALS AND METHODS

### 2.1 Philosophical stance

In the current study, a pragmatic process of inquiry was implemented by the authors, whereby the methods employed were perceived to be the most effective for addressing the research aims (Morgan, 2014). Both quantitative and qualitative methods were used to capture the perceptions of iMG managers in their immediate context via additional coverage (Morgan, 2014). Specifically, a quantitative approach was employed to measure and summarise iMG managers' agreement with specific statements relating to the research aims. Where practitioners' opinions were sought with respect to broader topics (open‐ended questions), a qualitative method (thematic analysis) was implemented as a process of identifying patterns within the answers the iMG managers provided.

### 2.2 Study design

A cross‐sectional survey design was used to investigate iMG managers' perceptions of the utility of iMGs in men's and women's rugby union and rugby league. Ethics approval was gained by the institutions Ethics Committee (114070) prior to data collection and informed consent was obtained for all participants prior to commencing the survey.

### 2.3 Participants

All iMG managers from rugby union (men's; Currie Cup [South Africa], Premiership [England], women's; Premier‐15s [England]) and rugby league (men's Super League [England], women's Super League [England]) competitions were eligible and invited to participate in the study. This included 21 men's rugby union (MRU), 12 men's rugby league (MRL), 10 women's rugby union (WRU) and 11 women's rugby league (WRL) iMG managers. The iMG manager was responsible for the implementation and day‐to‐day use of iMGs and associated systems within their practical setting.

### 2.4 Survey

An online survey was conducted using Google Forms from May to July 2023, a median of 3 (min = 3, max = 5) months after the iMGs had been implemented at the clubs. A web link was distributed to the iMG managers of all clubs eligible to participate via email. The survey instrument was developed by authors GR and BJ and sent to governing and non‐governing body representatives (LS, MC, EF, SK, KS, CR, DS and BJ). This was to ensure that the content was appropriate for acquiring specific information to help guide policymaking regarding iMG implementation globally and inform future research endeavours. Regarding content and face validity, items were discussed and agreed upon via group email and a live document with tracked changes and not quantified by way of content scoring and statistical analysis (e.g. content validity index). Additionally, the authors with iMG manager experience (GR, CO and SS) reviewed the wording of the questions to ensure appropriate interpretability for iMG manager participants (Arundel, 2023; Taherdoost, 2016). The survey consisted of 18 questions across four sections: (1) staff and player interest in iMG data, (2) the iMG technology, (3), use of iMG technology and (4) barriers to iMG use. Sections one to three consisted of five‐point Likert‐scales. Section three had an additional open‐ended question regarding iMG manager perceptions on what iMG devices are useful for. Section four consisted of two open‐ended questions regarding barriers to wearing the iMG devices and data engagement. All participants fully completed the survey.

### 2.5 Data analysis

Survey responses were exported from Google Forms and imported into R (version 4.3.0) and analysis was conducted using R Studio (Version 2023.06.1 + 524). Likert data were analysed using the likert() function of the likert package (version 1.3.5) (Bryer & Speerschneider, 2016) to produce bar charts for each question with bars centred on the middle response of the likert scale (‘neither agree nor disagree’ or ‘sometimes’). Qualitative data (i.e. open‐ended questions) were analysed via thematic analysis following Braun and Clarke's (2006) six‐phases: (1) familiarisation, (2) generation of codes, (3) searching for themes, (4) reviewing themes, (5) defining themes and (6) producing report. In addition, the 15‐point checklist provided by Braun and Clarke (2006) was used to ensure a rigorous and systematic process was followed throughout. An inductive thematic analysis approach was used to explore the practitioners' beliefs and identify patterns within the data. In phase one, the first author (GR) became familiar with the data and in phase two developed recurring features into initial codes. These codes were reviewed by a second author (SW) after her own familiarisation phase. In phase three, GR reviewed the codes and looked for broader patterns of meaning that were developed in preliminary themes. These were subsequently discussed and reviewed with SW in phase four. In phase five, GR refined the name and defined each theme which was reviewed by SW to ensure each theme had a coherent narrative. Common codes emerged across environments; therefore, groups were not split for reporting to provide richer data and support the development of themes.

## 3 RESULTS

A total of 46 out of the 54 (85%) iMG managers completed the survey with 20/21 (95%) from MRU, 9/12 (75%) from MRL, 7/10 (70%) WRU and 10/11 (91%) WRL.

### 3.1 Staff and player interest in iMG data

**FIGURE 1:**
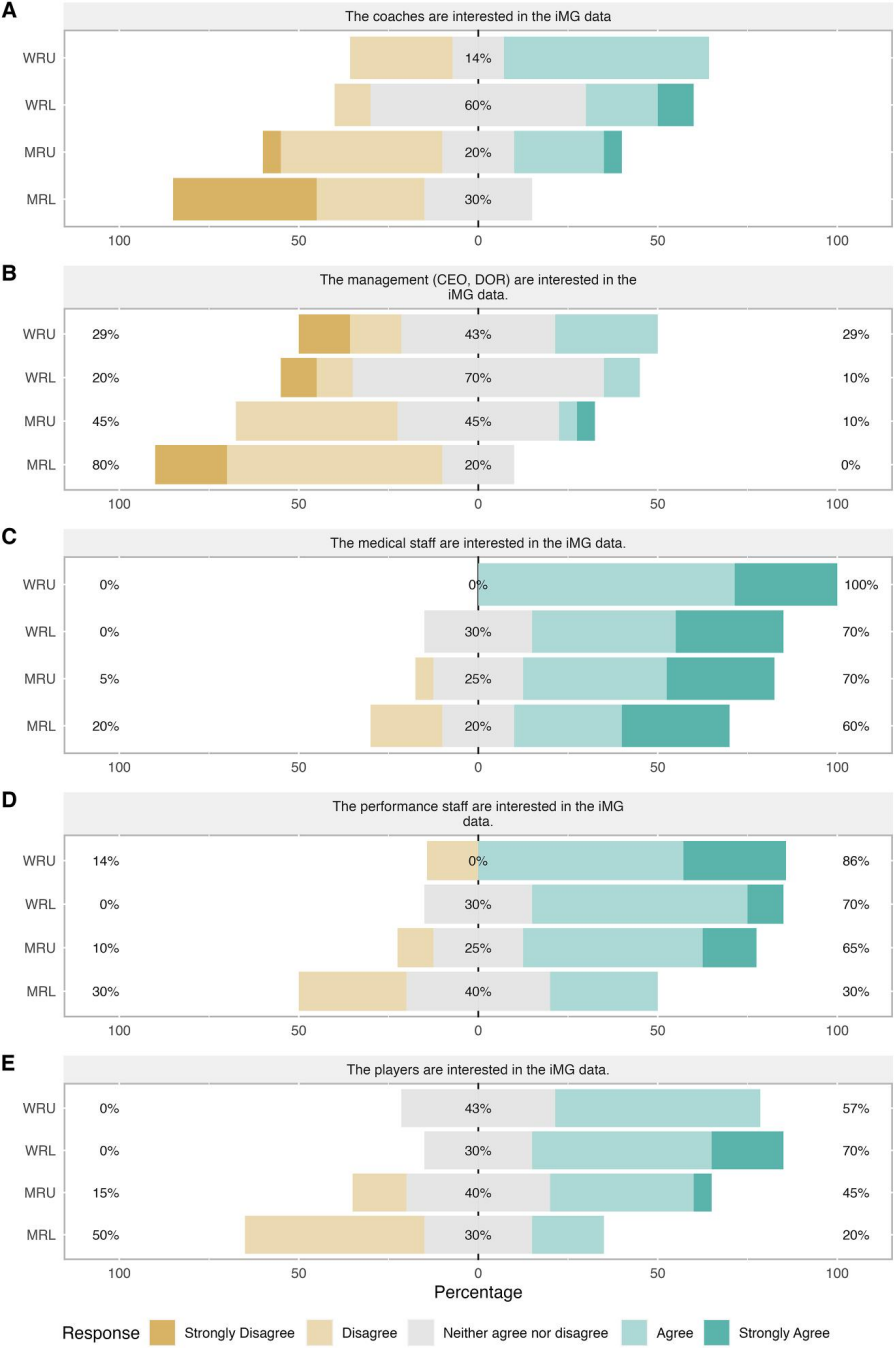
iMG manager responses to specific statements, A to E, regarding player and staff interest in iMG data. CEO, chief executive officer; DOR, director of rugby; iMG, instrumented mouthguard; MRL, men's rugby league; MRU, men's rugby union; WRL, women's rugby league; WRU, women's rugby union.

The iMG managers' responses to statements in section one regarding staff and player interest in iMG data are shown in Figure 1. When asked if ‘coaches are interested in iMG data’ 57% of WRU iMG managers' agreed, whilst in MRL and MRU, 70% and 50% disagreed/strongly disagreed (Figure 1A). Similarly, MRL and MRU iMG managers did not perceive management to be interested in iMG data (80% disagree/strongly disagree in MRL, and 45% disagree in MRU) (Figure 1B). In WRL, the majority of iMG managers neither agreed or disagreed that coaches or management are interested in iMG data (60% and 70% for coaches and management, respectively).

The majority (60%–100%) of iMG managers in all environments agreed/strongly agreed that medical staff are interested in iMG data (Figure 1C). In MRL, 30% of iMG managers disagreed and 30% agreed that performance staff are interested in iMG data. Whereas in MRU, WRL and WRU, most iMG managers (65%–86%) agreed/strongly agreed that performance staff are interested in iMG data (Figure 1D). The majority of WRL and WRU iMG managers perceived players to be interested in iMG data (57% and 70%, respectively). In MRU, 45% agreed/strongly agreed that players are interested in iMG data but 15% disagreed. In MRL, only 20% agreed with 50% of iMG managers disagreeing that players are interested in iMG data (Figure 1E).

### 3.2 iMG technology

**FIGURE 2:**
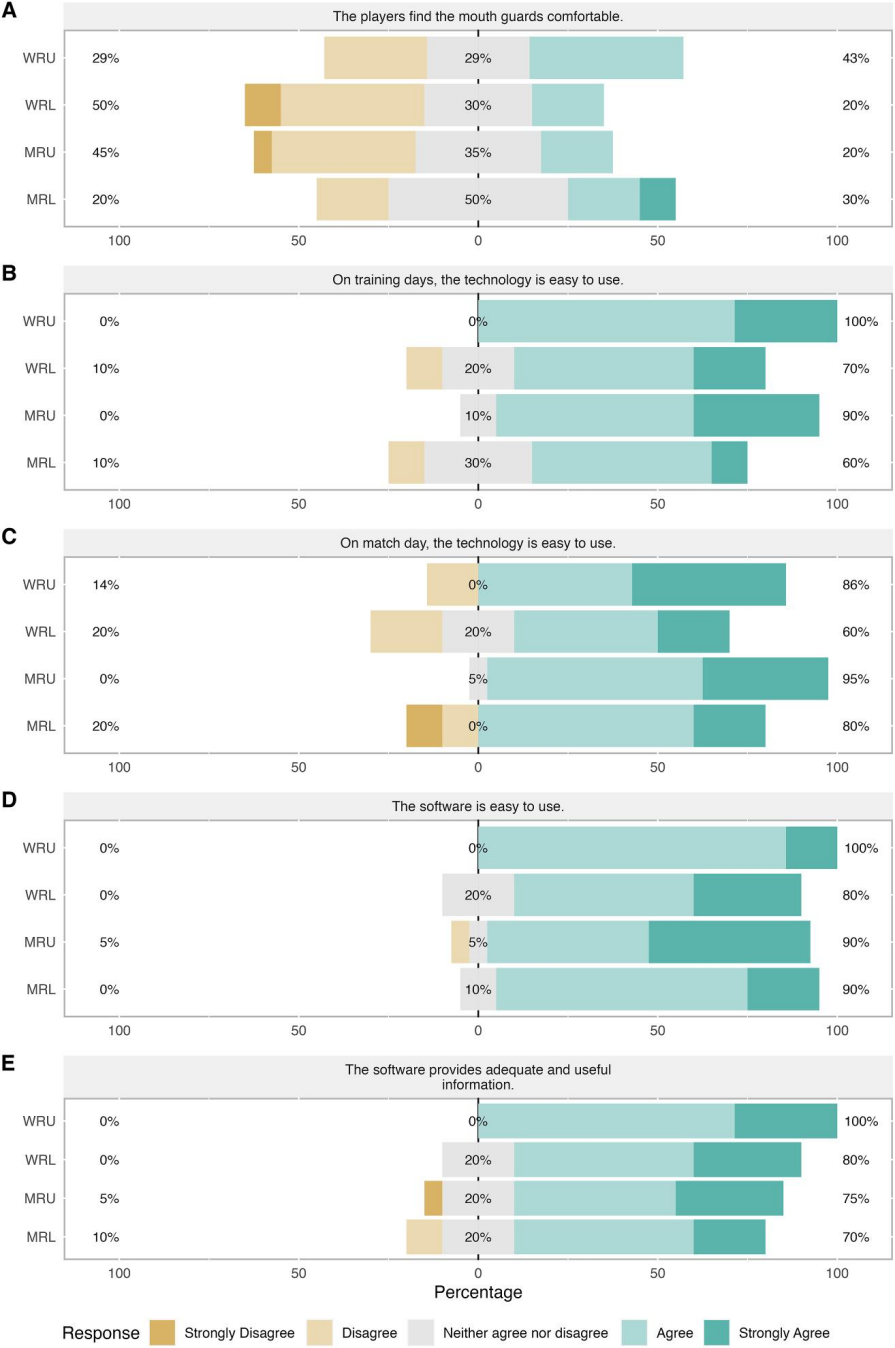
iMG manager responses to specific statements, A to E, regarding the technology. iMG, instrumented mouthguard; MRL, men's rugby league; MRU, men's rugby union; WRL, women's rugby league; WRU, women's rugby union.

The iMG managers' responses to statements in section two regarding iMG technology are shown in Figure 2. The responses from iMG managers to the statement ‘players find the mouthguards comfortable’ varied across all environments, 20%–50% disagreed/strongly disagreed, whilst 29%–50% neither agreed or disagreed and 20%–43% agreed/strongly agreed (Figure 2A). Most iMG managers agreed/strongly agreed that the technology was easy to use on training days (60%–100% [MRL and WRU]) and match days (60%–95% [WRL and MRU]) (Figure 2B,C). When asked about the software, the majority found it easy to use (80%–100% [WRL and WRU]) and that it provided adequate and useful information (70%–100% [MRL and WRU]) (Figure 2D,E).

### 3.3 Use of iMG technology

**FIGURE 3:**
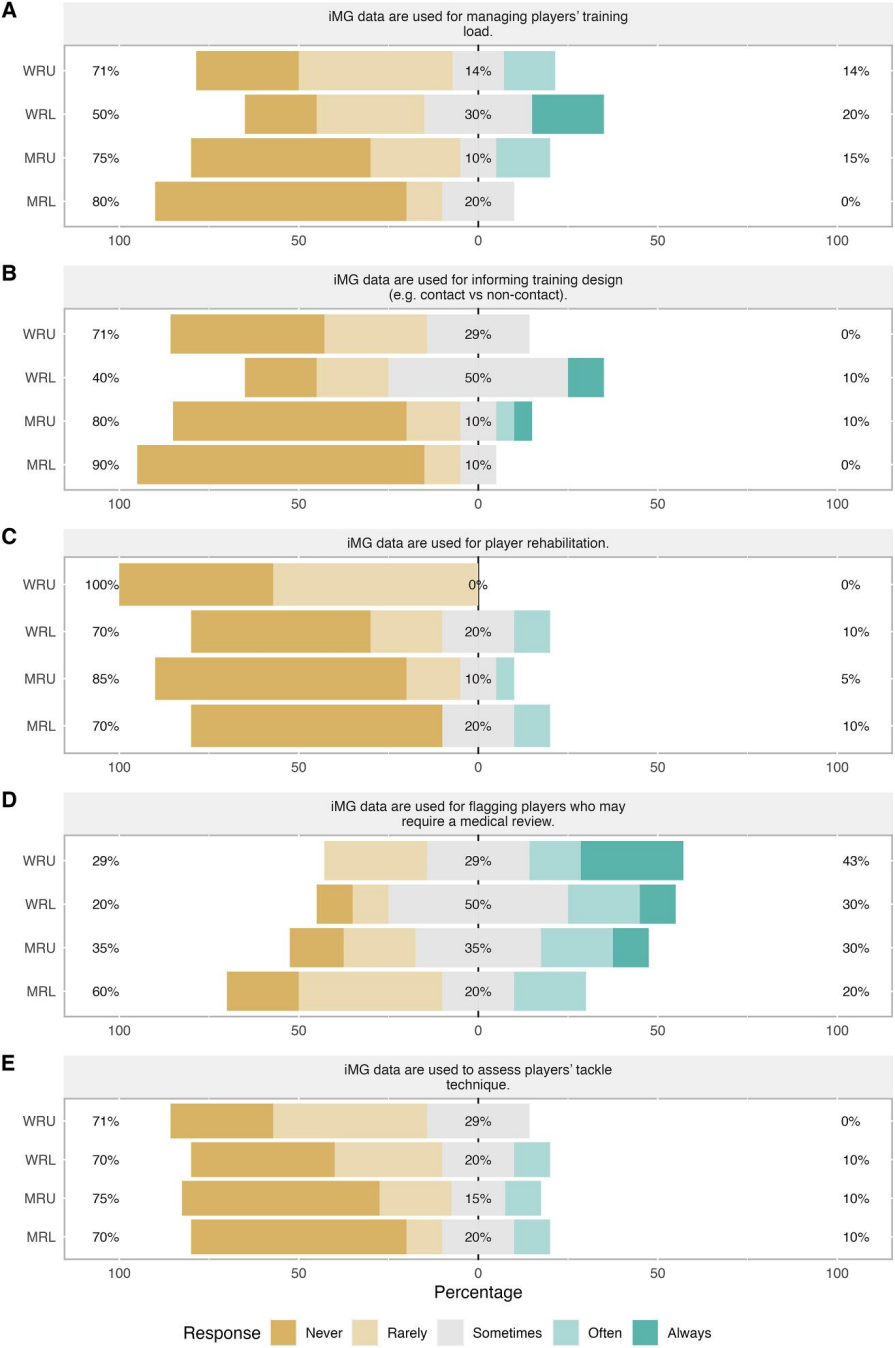
iMG manager responses to specific statements, A to E, regarding the uses of iMGs. iMG, instrumented mouthguard; MRL, men's rugby league; MRU, men's rugby union; WRL, women's rugby league; WRU, women's rugby union.

The iMG managers' responses to statements regarding the use of iMGs in section three are shown in Figure 3. The majority of iMG managers in WRU, MRU and MRL stated iMG data are never or rarely used for managing players' training load (71%–80%) or to inform training design (71%–90%). Whereas in WRL, 20% of iMG managers perceived iMG data to always be used to manage players' training load and 50% stated it is sometimes used to inform training design (Figure 3A,B). Across all environments, most iMG managers (70%–100%) perceived the data to be rarely or never used for player rehabilitation (Figure 2C). In WRU, 43% of iMG managers perceived data to be used to flag players for medical review, whilst in MRL, 60% of iMG managers stated it is never or rarely used in these circumstances (Figure 3D). Only 10% of iMG managers in WRL, WRU and MRL perceived iMG data to be ‘often’ used to assess players' tackle technique (Figure 3E).

When the iMG managers were asked ‘what are iMGs useful for?’, four themes emerged in the uses described: *player welfare*, *player monitoring*, *quantifying HAE in training and matches and planning training* and *medical applications*. The related codes for these themes and supporting quotes are shown in Table 1A. The most commonly occurring use was for medical applications particularly for identifying medical flags.

**TABLE 1:** Themes, their related codes and supporting quotes that emerged from responses to the open‐ended questions.

Themes	Descriptions	Codes	Quotes
(A) ‘In your opinion, what are iMGs useful for?’			
Player welfare	Participants communicated scenarios in which iMG data may be useful for identifying factors that contribute to HAE accumulation and brain injury	Tackle technique	‘Gives an indicator to check on players who might have high loads or big impacts focusing on technique flaws’.
		Identifying risk factors	‘Protection for players, a sense of confidence that the research will lead to great things in the future for the game, understanding potential present and future head related issues better’.
		Reduce concussion	
		Player protection	‘…reducing the risk of concussion and enable the analysis of factors that may contribute to this increased risk’.
Quantifying HAE in training and matches for planning and monitoring	The iMG managers explicitly described the usefulness of iMG data to individually monitor a player's exposure to HAEs and implied their use for guiding individual training load adjustments. Participants also referred to iMGs as a tool for quantifying HAEs during training and match‐play and implicitly suggested using this data to guide general periodisation practices	Player monitoring	‘…monitoring contact load to allow any modifications to take place if necessary…’.
			‘We have found them very useful when monitoring player load, particularly post game, when looked at with other performance measures’.
		Training session planning	‘I think it could be very useful when planning training to understand which drills elicit a high head impact workload and tailoring training in order to minimise potentially effects of this when playing on the weekend’.
		Understanding HAE content of training activities	
		Provide data on HAE	
		Quantifying HAE in training and matches	‘Quantification of match and training demands’.
Medical applications	Participants described contexts in which medical practitioners could implement iMG technology to augment their practice	Medical flag	‘Measuring impacts and alerting medical staff to be aware of the impacts and assessing the player further’.
		Graded return to play	‘iMGs have been extremely useful in identifying high impact alerts in training and game…contact re‐load after a HIA has also been aided through the use of individual player loads’.
		Medical	
(B) ‘In your opinion, what are the major barriers to players wearing the iMG mouthguards in training and matches?’			
The comfort, size and fit of the iMG mouthguard	The iMG managers explained on how the shape and physical mass of the iMGs influenced players' perceptions of comfort	Comfort	‘The shape, fit and bulkiness of the devices’.
		Size	‘Players don't find the fit comfortable due to the length they go back and how thick they are at the front’.
		Fit	
Inconsistent use of un‐instrumented mouthguards	Participants communicated instances where players did not wear regular (un‐instrumented) mouthguards and implied that this behaviour continued when iMGs were introduced	Preference for own mouthguard	‘Not wanting to always wear a mouthguard in training sessions’.
		Mouthguards only worn in contact training	‘…some guys actually don't like playing with a mouthguard’.
		Some players don't wear mouthguards	‘An issue sometimes with wearing during training is that if the player perceives there to be little/limited contact in the session, they may not pick up their mouthguard’.
		Some players don't wear mouthguards in training	
		Some players don't wear mouthguards in matches	
Negative impact on performance	Participants illustrated scenarios in which players perceived wearing an iMG to directly (wearing an iMG impairs their ability to perform) or indirectly (iMG data is used to influence selection) affect their performance in a negative way	Fear of being side‐lined	‘…players not wanting to wear for games due to communication issues’.
		Impairs communication	‘Some players also feel that wearing a mouthguard restricts their on field performance’.
		Impairs performance	‘…concerns that their data may be used in a way that will stop them playing…’
(C) In your opinion, what are the major barriers to players and staff engaging with iMG data?’			
Lack of understanding of what the data means	The iMG managers explicated a lack of knowledge with respect to how the data could be used to inform practice, suggesting an absence of scientific or comparative data as a major contributing factor	Understanding of the data	‘Lack of understanding on how the data can be used/what the data actually means in terms of clinical outcome’.
		Limited evidence available to inform decision‐making	‘Besides knowing a huge impact happened over 40 g, there is no guidelines for mid‐range impact, so it is hard to plan/reflect/change for contact sessions’.
		Lack of key benchmark data	
Time constraints	Finding adequate time in the working day to engage with the iMG data was perceived as a major barrier by participants	Insufficient time	
		Part‐time medical staff	‘Lack of time as an MDT (other things are a priority for staff before the use of this technology)’.
Players not consistently wearing the iMGs	Participants discussed situations where players did not regularly wear iMGs, and either implicitly or explicitly suggested that this compromised the usefulness of the data	Players do not consistently wear the devices	‘Staff time and availability doing other daily tasks’.
		Player buy‐in	‘Players buying into their usefulness. Players habits around the use of a mouthguard’.
Technology issues	Issues with proper functioning of the iMG hardware and resulting untrustworthiness of the data produced were presented as major barriers to staff and players engaging with the iMG data	Technology failures	‘Inconsistency of use from players—If iMGs aren't worn consistently (which was common) any training load monitoring becomes difficult’.
		Lack of trust in the technology/data	‘The mouthguards are not reliable enough as they keep breaking, so it is difficult to get an accurate loading picture’.
		Lack of confidence in the data	‘There are times as well with some high G impacts that values have appeared to be unusually high for a non‐contact session to the point where sometimes I don't trust the data’.
		Usability and reliability of data	

Abbreviations: HAE, head acceleration event; iMGs, instrumented mouthguards.

### 3.4 Barriers to iMG use

In response to ‘what are the major barriers to players wearing the iMG mouthguards in training and matches?’, three themes emerged: *the comfort, size and fit of the mouthguard*, *inconsistent use of un‐iMGs* (e.g. players who sometimes do not wear a mouthguard during training or matches) and *negative impact on performance*; see Table 1B for related codes and supporting quotes. The *comfort* of the mouthguard specifically was the most identified issue by the iMG managers in all environments.

Four themes emerged from the iMG managers' responses to barriers to players and staff engaging with iMG data: *lack of understanding of what the data means*, *time constraints*, *players not consistently wearing the iMGs* and *technology issues*. The related codes for these themes and supporting quotes are shown in Table 1C. The iMG managers' frequently highlighted time constraints to engage with the data within their environment as well as a lack of understanding of what the data mean as barriers to engaging with the data.

## 4 DISCUSSION

This study aimed to investigate iMG managers' perspectives on the interest and use of iMG technology within men's and women's rugby league and union. Secondly, it aimed to identify their perceived barriers to implementation, to support future adoption. The players and staff interest in iMG data, as perceived by the iMG managers, varied for different staff roles. However, several uses of the data were identified and barriers to use were highlighted. These included *time constraints* and a *lack of understanding of the data*. The iMG managers perceived the iMG devices to be easy to use, but that the players found the devices uncomfortable with the *comfort, size and fit of the iMG* reported as a barrier to wearing the devices. In addition, *inconsistent use of un‐instrumented mouthguards*, and *perceived negative impacts on performance* were also reported as barriers to players wearing the mouthguards.

The primary interest and the use of iMG data, as perceived by the iMG managers in the current study, was medical. The majority of iMG managers (60%–100%) perceived medical staff to be interested in iMG data, compared to the high percentages either disagreeing, or neither agreeing nor disagreeing, that coaches and managers were interested in the data (Figure 1). This is further supported by 40%–80% of iMG managers across environments stating the data was currently ‘sometimes’ to ‘always’ used for flagging players for medical review. Furthermore the themes of *medical applications* and *player welfare* emerged when practitioners were asked what iMG devices are useful for. This could be due to how the technology was embedded within teams, with implementation and education directed through the medical staff, particularly in rugby union. A high proportion of iMG managers also perceived the performance staff to be interested in iMG data, which is again in line with other emerging themes of player monitoring and quantifying HAE in training and matches and planning training. However, performance staff need to work as a multi‐disciplinary team with the coaches to implement changes based on these data. Thus, to ensure iMGs make an impact in practice, focus should be on increasing coaches' interest in iMG data and developing an aligned strategy within the organisations (Fullagar et al., 2019) to increase use of data as well as player buy‐in.

The players' interest in iMG data, as perceived by the iMG managers, varied across the different environments, but 20%–60% agreed/strongly agreed that players were interested (Figure 1E). However, despite perceived interest, anecdotally, it is evident that the use and uptake of iMGs varies. The current study has identified several reasons and barriers for this. A large percentage of iMG managers disagreed with the statement that ‘players find the mouthguards comfortable’ (Figure 1). Moreover, when the iMG managers were asked what they perceived the major barriers to players wearing the iMG devices to be, *the comfort, size and fit of the iMG mouthguard* emerged as a theme across the responses (Table 1B). This highlights the need to make the devices more comfortable to increase adoption. Additionally, the preference of players not to wear any mouthguard, particularly in training, or having preference to wear their own, were identified as barriers to player wearing the iMG devices (*inconsistent use of un‐instrumented mouthguards*; Table 1B). Furthermore, iMG mangers reported that players who do wear the iMG devices, do not wear them consistently across matches and training, which acts as a major barrier to use of the data (*players not consistently wearing the iMGs*; Table 1C). This is in agreement with mouthguard literature (Boffano et al., 2012; Ilia et al., 2014; Rayner, 2008) with one study in rugby union reporting only 54% of players wear their mouthguard in all training and matches (Boffano et al., 2012). Wearing mouthguards at a younger age has been associated with wearing the mouthguard as an adult in Japanese rugby union players (Hayashi et al., 2020); therefore, introducing the iMG devices at a younger age could increase compliance with wearing the devices. However, given the final barrier that emerged of a perceived *negative impact on performance* with *fears of being side‐lined* (Table 1B), continued education is required across all levels of competition to promote their use and importance. Alternatively, governing bodies may mandate mouthguards and/or iMGs given their potential for player welfare.

The iMG technology was perceived by the majority of iMG managers as being simple to use on training and match days with easy‐to‐use software that provides adequate and useful information (Figure 1). Despite this, a major barrier to engaging with iMG data was *time constraints* with ‘other things…a priority for staff before the use of this technology’. Given that a *lack of understanding of what the data means* emerged as another major theme in the barrier to engaging with iMG data, it could be argued that by developing the body of research to provide benchmark data and evidence to inform decision‐making (Table 1C), practitioners could prioritise time to engage with iMG data, particularly if the coaches were interested. The recent widespread distribution of iMG devices within the rugby codes will support the growth of research in the area providing practitioners with a greater understanding of what the data means. However, increased uptake of the devices by players is firstly required to ensure high‐quality research that can inform decision‐making (Abt et al., 2020).

Overall, the iMG manager responses appeared similar between rugby codes and sexes. However, of note, the iMG managers perception that coaches and players were interested in the iMG appeared to differ between sexes with greater interest reported in both female rugby league and union compared to the male codes (Figure 1). In both rugby codes, the men's game is professional, while the women's game is primarily amateur. Thus, it is possible that the professional teams already had a number of different technologies in use and the addition of iMG technology did not provide perceived novelty or benefit. Furthermore, iMG managers working in men's rugby league provided the lowest agreement for all statements regarding staff groups and player interest in the data. It is difficult to hypothesise why this was the case and future research should be undertaken to help elucidate these findings.

Whilst this study provides important contextual information and identifies barriers to the adoption of the iMG devices, only the iMG managers were surveyed. Although they are the closest to the devices from a day‐to‐day use perspective, their perceptions may be different from the players and other staff members. Furthermore, it was likely difficult for iMG managers to be passive observers in this research as they were embedded in the context in which the technology was being implemented. Thus, their perceptions may have been biased by the strength and quality of their relationships with other staff and players in addition to their own opinions about the technology. Therefore, future research needs to interview players and other stakeholders specifically. Additionally, the role of the iMG manager within the team (i.e. if they were already embedded strength and conditioning staff vs. newly appointed staff or interns) could have influenced the ability to drive buy‐in from other staff and players and should be considered when implementing adoption strategies. Furthermore, although the survey was designed by relevant domain experts to enhance content and face validity, quantitative validation of the instrument was not undertaken. Moreover, no reliability testing for the instrument was carried out. Therefore, it is possible that iMG manager perceptions may not have been stable over time or been fully captured. As such, replication studies may be required to ensure findings accurately represent iMG manager perceptions. Finally, compliance data are needed to support the findings and understand when and by whom the mouthguards are worn to provide targeted strategies and education to increase their use.

## 5 CONCLUSION

This current study provides an insight into the current interest and use of iMG technology and the barriers to implementation of the devices. Since the widespread distribution and implementation of iMG devices across the environments investigated (elite male and female, rugby union and league), anecdotally, the uptake and use of the devices has varied by the team. Therefore, this study provides data which could support strategies to improve their uptake and use. The findings suggest continued education, or mandating iMGs, alongside improving the fit and comfort of the iMG devices, may increase player and staff buy‐in. Developing the body of research will help support practitioners in understanding the data and making informed decisions, therefore making iMG data more useful and increasing engagement. However, to do so, players must be wearing the devices. Thus, it could be proposed that the priority is overcoming barriers to players wearing mouthguards and also educating coaches to influence player buy‐in.

## CONFLICT OF INTEREST STATEMENT

There are no conflicts to declare.
